# Anatomic Restoration of Triple Disruption of the Superior Shoulder Suspensory Complex: A Case Report and Review of the Literature

**DOI:** 10.1111/os.12764

**Published:** 2020-09-25

**Authors:** Kai Wu, Xiao‐ming Wu, Xiao‐long Zha, Qiu‐gen Wang

**Affiliations:** ^1^ Department of Trauma Orthopaedics Shanghai General Hospital Affiliated to Jiaotong University Shanghai China

**Keywords:** Triple, Disruption, Superior shoulder suspensory complex, Restoration

## Abstract

**Background:**

Multiple disruptions of the superior shoulder suspensory complex (SSSC) involving more than two components are extremely rare. In some extreme situations, three components of the SSSC structure can be involved. The ideal treatment for this type of injury is debatable.

**Case presentation:**

A 21‐year‐old woman was referred to our emergency center following a traffic accident. A three‐dimensional CT scan showed triple disruption of the SSSC involving concomitant ipsilateral fractures of the coracoid, the acromion, and the distal clavicle. The connection between the upper limber and the axial skeleton was destroyed. There was no evidence of associated injury and the neurovascular examination of the injured upper limb was normal. The patient underwent an open reduction and internal fixation to restore the anatomic integrity of the SSSC. The arm was supported in a broad arm sling for 2 weeks after surgery. Gentle passive range of motion activity under analgesic was encouraged from the second day postoperatively. One year and half after the operation, the patient had regained pain free and unrestricted shoulder stability and mobility.

**Conclusion:**

The manifestations of multiple disruptions of the SSSC may be variable. This case illustrated the challenges of treating the multiple disruption of the SSSC structure. It also showed that surgical intervention for this rare combination injury yields an excellent functional result. The good outcome achieved in this patient demonstrates that surgical intervention might be an optional resolution for multiple disruptions of the SSSC.

## Graphical abstract

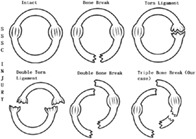

The SSSC is a ring of bone and soft tissue, which is composed of the coracoclavicular ligament, the distal clavicle, the AC joint, the coracoacromial ligament and the acromion, the coracoid process, and the glenoid. We presented a case of a triple bone break of this ring.

## Background

Isolated coracoid fractures and acromion fractures are rare^1^, ^2^. Combined coracoid and acromion fractures, which are usually referred to as double disruptions of the SSSC, are even more uncommon^3^. However, in some extreme situations, multiple disruptions of the SSSC involving more than two components of the SSSC structures can occur. Only a few case reports have described this type of injury^4^, ^5^, ^6^, ^7^, ^8^. The SSSC is a ring of bone and soft tissue, which is composed of the coracoclavicular ligament, the distal clavicle, the AC joint, the coracoacromial ligament and the acromion, the coracoid process, and the glenoid. The ring is further subdivided into three units: (i) the clavicular–AC joint–acromial strut; (ii) the three‐process scapular body junction; and (iii) the clavicular–coracoclavicular ligamentous–coracoid linkage (C4 linkage). Lesions to two of these structures cause significant displacement to occur at the individual site and for the entire SSSC.

If two of the three fractured parts are combined, this will compromise one of the three sub‐SSSC rings. The acromion fracture, which descended into the spinoglenoid notch, combined with the coracoid base fracture compromised two components of the three‐process scapular body junction. The distal clavicle fracture with the acromion fracture disrupted the superior strut of the SSSC. The coracoid fracture combined with the distal clavicle fracture violated the C4 linkage integrity. The injury pattern presented here belonged to the multiple‐ring disruption, which we refer to as “triple disruption” of the SSSC.

The ideal treatment for double disruption of the SSSC is controversial. Conservative treatment of the floating shoulder has been reported to be as successful as operative management^9^, ^10^. Proponents of the latter believe that stabilizing one or two of the involved components would enforce shoulder stability by restoring the integrity of the shoulder girdle. However, controversy remains about whether to just restabilize one component of the disrupted structures or to restore the integrity of all the violated components^11^.

## Case Presentation

### *Clinical Presentation and Imaging Findings*

**Fig. 1:**
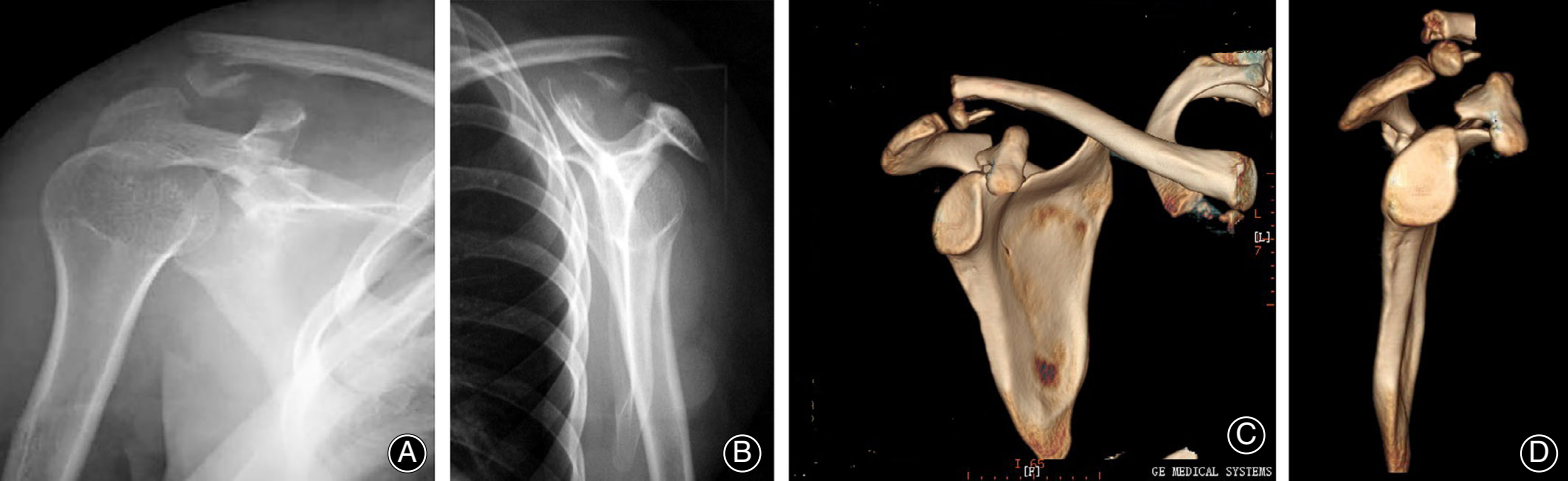
(A, B) Anteroposterior and transscapular view of the right shoulder demonstrating the distal clavicle, acromion, and coracoid fractures; (C–D) three‐dimensional reconstructions showing the inferiorly displaced distal part of the clavicle fracture, the proximal part of the clavicle migrated superiorly with the coracoid base, and the acromion or the lateral scapular spine fracture extending into the spinoglenoid notch.

A 21‐year‐old woman was struck by a car from behind while riding a bicycle and was referred to our emergency center. The clinical examination showed loss of normal shoulder contour and mild bruising around the right scapular spine. There was no evidence of associated injury and the neurovascular examination of the injured upper limb was normal. Shoulder radiographs revealed fractures of the coracoid process, the acromion, and the distal clavicle (Fig. 1). Further three‐dimensional CT reconstruction revealed a minimally displaced acromion fracture extending into the spinoglenoid notch, a displaced coracoid base fracture, and a distal clavicle fracture (Fig. 1). The coracoclavicular distance was normal. Considering the patient's age and the radiographic evidence of significant fracture displacement, the decision to perform surgery was made after discussing the treatment options and risks with the patient.

### *Surgical Procedure*

**Fig. 2:**
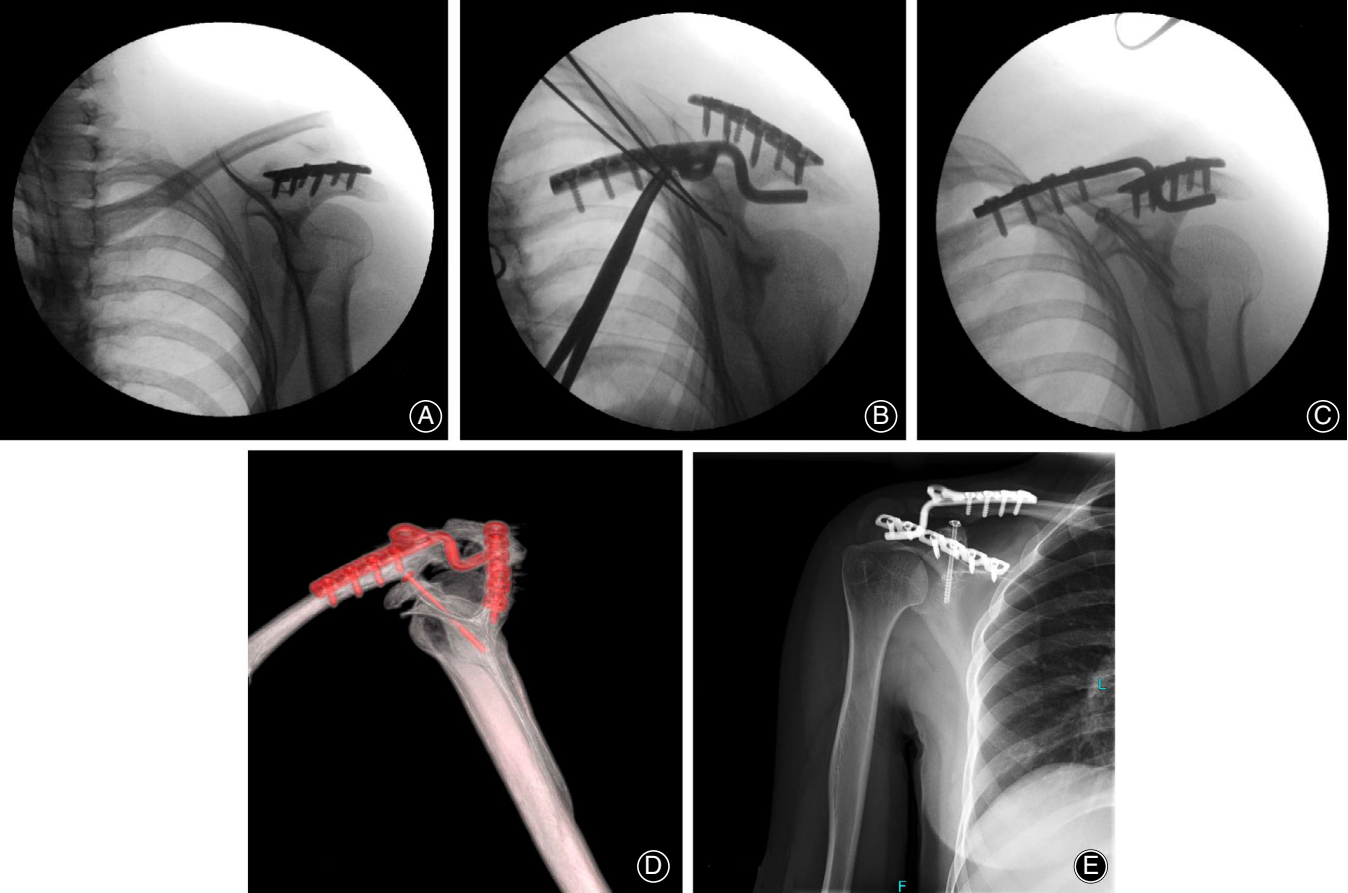
(A) Reduction and fixation of the acromion fracture with a 3.5 mm reconstruction locking plate; (B) placement of the clavicular hook plate; (C) insertion of the cannulated lag screw along the guide wire; (D) postoperative three‐dimensional CT showing the anatomic reconstruction of the disrupted superior shoulder suspensory complex (SSSC); (E) anatomical fracture healing and intact hardware 2 years postsurgery; there is erosion under the acromion due to the hook plate.

Surgery was performed under general anesthesia 2 days after the injury. The patient was placed in the beach‐chair position with the injured limb draped free for movement during the operation. The acromion fracture line extended to the scapular spine. First, an 8‐cm incision was made along the scapular spine. After retraction of the posterior deltoid laterally, the acromion fracture was exposed. It was located at the junction of the acromion base and the lateral scapular spine, extending inferiorly into the spinoglenoid notch with minor displacement. The acromion fracture was fixed with a 3.5‐mm reconstruction locking plate (Trauson, Changzhou, China) (Fig. 2). A second incision was made along the distal clavicle extending 2.0 cm lateral to the AC joint, after which the distal clavicle and the AC joint were exposed. The lateral part of the clavicle fracture was connected with the acromion by the intact AC capsule. After reduction of the clavicle fracture, a 2.0‐mm Kirschner wire was used for temporary fixation. The AC joint remained untouched. Then, the clavicular hook plate (Trauson, Changzhou, China) was applied. The hook was inserted under the acromion to elevate the acromion and the plate was placed along the clavicle to depress the migrated clavicle (Fig. 2). The coracoid base fracture was addressed percutaneously. The entry point at the junction area of the superior and inferior coracoid pillar was identified and the guide wire was advanced under fluoroscopic guidance through the coracoid base to the scapular neck. After the guide wire position was confirmed, the depth of the guide wire was measured. A 4.0‐mm cannulated titanium screw (Trauson, Changzhou, China) was inserted along the guide wire and the coracoid base fracture was fixed (Fig. 2).

### *Postoperative Management*

Postoperatively, the arm was supported in a broad arm sling for 2 weeks. Gentle passive range of motion activity under analgesic was encouraged from the second day postoperatively. The rehabilitation program consisted of Codman exercises and passive shoulder range of motion exercises for 4 weeks, followed by active range of motion exercises, which progressed to resistive exercises after 6 weeks. The patient was instructed to restrict her active elevation below 90° until the hook plate was removed, 5 months after the operation.

### *Final Follow‐up Result*

However, the patient was lost to further follow‐up. One and a half years later, she returned with an asymptomatic shoulder with a full range of motion. The final Constant–Murley score was 82. X‐rays showed that the fracture had healed in anatomical position and the hardware was intact (Fig. 2). Her only complaint was occasional discomfort around the AC joint. The hook plate was removed in another hospital. The postoperative X‐rays showed evidence of acromion erosion around the hook tip.

## Discussion

**Fig. 3:**
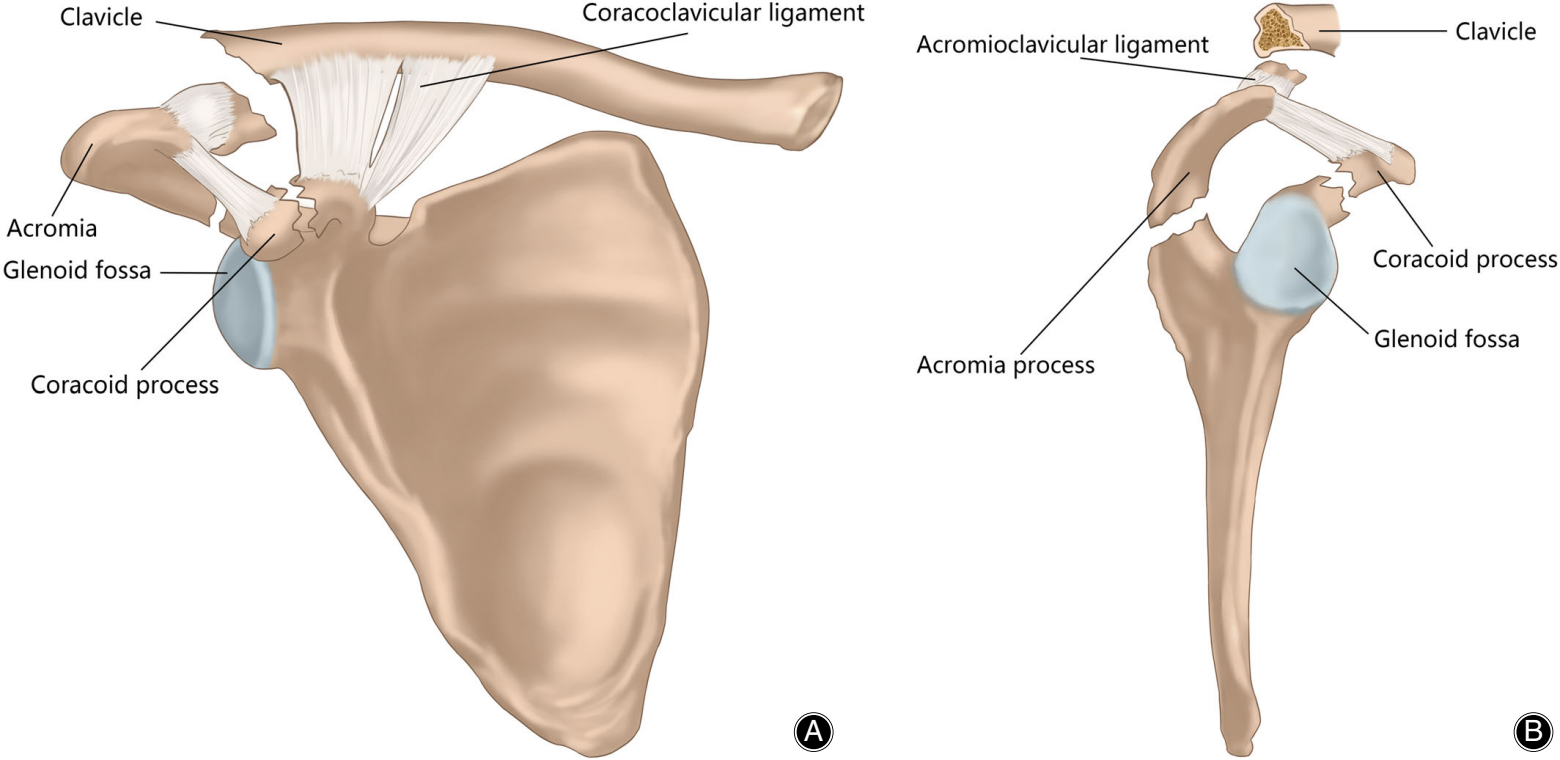
Illustration depicting the superior migration of the medial part of the clavicle fracture with the intact coracoclavicular ligament. The distal clavicle fracture locates lateral to the coracoclavicular ligament is a Neer type‐I fracture but is functionally equivalent to a Neer type–II distal clavicle fracture. The tip of the coracoid is displaced inferiorly and laterally with the pull of the conjoined tendon.

Coracoid fractures are uncommon, occurring in approximately 2% to 5% of all scapular fractures, with scapular fractures, in turn, accounting for only 1% of all fractures. Most of the coracoid fractures occur at the base and are part of a complex shoulder girdle injury^12^. Eyres classified the coracoid fracture into five types according to their anatomical position^13^. It was further subgrouped into A or B, according to whether the AC joint was dislocated or not. This classification system emphasizes the importance of the integrity of the scapuloclavicular connection. If the bony insertion of the coracoclavicular ligament on the coracoid has been preserved, the fractures respond well to conservative treatment (Eyres I–IIIB). If the fracture extends into the coracoid base, the connection between the clavicle and the scapula is destroyed and surgical management is recommended (Eyres IIIA–V). In this case, the fracture at the coracoid base without AC joint dislocation belonged to the Eyres IIIB coracoid fracture group. According to Eyres *et al*., this type of isolated coracoid fracture, displaced or not, can be successfully treated by conservative means. However, this is not so in the presence of a concomitant distal clavicle fracture. According to the Neer classification, a distal clavicle fracture located lateral to the coracoclavicular ligaments is Neer type‐I fracture. With the coracoclavicular ligament intact, it should be inherently stable, with no or minimal displacement, and respond well to conservative treatment. However; it is different when the coracoid and the clavicle are fractured concomitantly. The coracoclavicular ligament lost its bony insertion on the coracoid. The medial part of the clavicle fracture and the coracoid process migrated superiorly as a whole, while the distal part of the clavicle was displaced inferiorly (Fig. 3). The scapuloclavicular connection was destroyed. The most common type of scapuloclavicular disconnection associated with a coracoid process fracture is ipsilateral AC joint separation. Although it is uncommonly encountered in daily practice, this type of AC joint dislocation has been described by several authors^14^, ^15^. The present case involves the distal clavicle fracture instead (Fig. 3). We found only a few case reports describing this injury pattern^16^. Ogawa demonstrated a case series of 15 chronic coracoid fractures, of which only one Ogawa type‐I coracoid fracture was associated with a distal clavicle fracture^12^. In a series of 12 coracoid fractures reported by Eyres, one patient sustained a coracoid fracture extending into the superior border of the scapula and the glenoid, with an ipsilateral displaced clavicle fracture^13^. This type of scapuloclavicular disconnection can also be termed the double disruption of the SSSC. Goss felt that the Neer type‐I distal clavicle fracture with the coracoid fracture was functionally equivalent to the unstable Neer type‐II distal clavicle fracture, and should be treated as such. Compared with reports of treatment of double disruptions of the SSSC, the literature on multiple disruptions of the SSSC is limited. To our knowledge, the surgical strategies are poorly documented. Goss reported two cases similar to ours^17^. In the first case, the acromion fracture was simply stabilized with a tension‐band construct, while the distal clavicle fracture and the coracoid process fracture were not addressed surgically. The fractures reduced spontaneously. In the second case, the non‐displaced acromion fracture was fixed with a plate; the distal clavicle fracture was stabilized with the tension‐band construct and the coracoid process fracture with a Kirschner wire. The bone healed in both cases. Goss later recommended the use of a cannulated screw instead of a Kirschner wire for coracoid fractures. Eyres *et al*. reported the case of a displaced clavicle fracture associated with a coracoid fracture extending into the superior border of the scapula and the glenoid. They fixed the coracoid process fracture using a single large‐fragment 3.5‐mm cancellous screw inserted through the base of the coracoid into the body of the scapula. The clavicle was fixed with a six‐hole plate. However, Eyres did not describe the surgical sequence. Lecoq presented a case similar to ours^4^. A 29‐year‐old patient sustained a displaced fracture of the coracoid process associated with a displaced acromion fracture and an undisplaced distal clavicle fracture. Only fixation of the coracoid fracture with a screw was performed, but the acromiohumeral distance was decreased to 5 mm postsurgery. In 2020, Yao reported 22 multiple SSSC injury cases (including 7 cases of triple SSSC injury) treated with ORIF. The final outcomes showed that ORIF is reliable for treatment of multiple injuries of the SSSC. Combined with active postoperative rehabilitation program intervention, it can accelerate the recovery of shoulder joint function^18^.

As no consensus exists on the optimum treatment strategy for triple disruptions of the SSSC, it appears that the treatment of multiple lesions of the SSSC should be individualized. Considering her age and the shoulder instability, surgical treatment seemed to be the only option for our patient. We started the surgical procedure with the scapular spine and then proceeded with the distal clavicle, because this approach was technically easy to access and to manipulate. The superior strut of the SSSC would be stabilized if the clavicle fracture and the acromion fracture were addressed properly. We first reduced and fixed the acromion fracture with the 3.5 mm reconstruction locking plate. The clavicular hook plate was applied later. In this case, the clavicular hook plate was chosen because it acted like a lever arm, elevating the acromion and depressing the clavicle, the latter being related to the spontaneous reduction of the superiorly migrated medial clavicle fracture.

The necessity for further surgery for the coracoid base fracture in this case can be discussed. We used a cannulated lag screw to fix the coracoid fracture under fluoroscopy for the reason that the reinserting the bony attachment of the coracoclavicular ligament would reduce the pressure generated by the clavicular hook tip under the acromion, the main reason for acromion erosion^19^.

In this case, we chose to fix all the fracture fragments to restore the integrity of the shoulder girdle. Skeletal stability was achieved, allowing for early shoulder motion and rehabilitation. The clinical outcome in our patient was excellent. After 2 years of follow up, the only adverse event was acromion erosion, due to retention of the hook plate^9^, ^19^. This can be readily resolved when the hook plate is removed^9^.

### *Conclusion*

The purpose of this case report was to demonstrate that the manifestations of multiple disruptions of the SSSC may be variable. The unexpected complexity of the injuries could have led to subtle but important findings being overlooked. This case highlights the importance of a thorough search strategy, consideration of injury biomechanics, and knowledge of associated injuries^20^. The successful outcome achieved in our patient demonstrates that operative treatment might be an optional resolution for multiple disruptions of the SSSC. The decision of whether to restore the integrity or just restabilize part of the broken structures should be tailored to the individual patient. The present study shows that surgical intervention for this rare complex injury can yield an excellent functional result.

## Author Contributions

The first author, Dr K. Wu, wrote this paper. Dr X. Zha and Dr Q. Wang participated in the operations. The corresponding author, Dr X. Wu, revised the paper.
